# Chronic Traumatic Encephalopathy: A Review

**DOI:** 10.1155/2012/816069

**Published:** 2012-04-10

**Authors:** Michael Saulle, Brian D. Greenwald

**Affiliations:** ^1^Touro College of Osteopathic Medicine, 230 West 125th Street, New York, NY 10027, USA; ^2^Brain Injury Rehabilitation, Mount Sinai Hospital, 5 East 98th Street, Box 1240B, New York, NY 10029, USA

## Abstract

Chronic traumatic encephalopathy (CTE) is a progressive neurodegenerative disease that is a long-term consequence of single or repetitive closed head injuries for which there is no treatment and no definitive pre-mortem diagnosis. It has been closely tied to athletes who participate in contact sports like boxing, American football, soccer, professional wrestling and hockey. Risk factors include head trauma, presence of ApoE3 or ApoE4 allele, military service, and old age. It is histologically identified by the presence of tau-immunoreactive NFTs and NTs with some cases having a TDP-43 proteinopathy or beta-amyloid plaques. It has an insidious clinical presentation that begins with cognitive and emotional disturbances and can progress to Parkinsonian symptoms. The exact mechanism for CTE has not been precisely defined however, research suggest it is due to an ongoing metabolic and immunologic cascade called immunoexcitiotoxicity. Prevention and education are currently the most compelling way to combat CTE and will be an emphasis of both physicians and athletes. Further research is needed to aid in pre-mortem diagnosis, therapies, and support for individuals and their families living with CTE.

## 1. Introduction

Chronic traumatic encephalopathy (CTE) has been defined as a progressive neurodegenerative disease caused by repetitive head trauma [1]. CTE was first described in 1928 when Dr. Harrison Martland, a New Jersey medical examiner, began to note a constellation of symptoms in boxers. In an article he published in the Journal of the American Medical Association entitled Punch Drunk, he describes the boxers, “cuckoo,” “goofy,” “cutting paper dolls,” or “slug nutty” [2]. Punch drunk was later termed dementia pugilistica, literally meaning dementia of a fighter. However, with the evolution of sports like American football, these symptoms were also being reported in athletes other than boxers and was renamed chronic traumatic encephalopathy in the 1960s.

 CTE has become a popular topic due to its close association with American football, hockey, soccer, boxing, and professional wrestling. Many of these affected athletes, mostly retired, have struggled in their later years with depression, substance abuse, anger, memory/motor disturbances, and suicide [3]. Autopsy results from these athletes have suggested a link between these emotional, cognitive, and physical manifestations and CTE [3–5]. In addition to athletes, military soldiers have become another group of interest as many are returning from the battlefield with brain injuries from blast trauma causing closed head injury. In this paper we present a summary of the epidemiology, risk factors, clinical presentation, pathophysiology, neuropathological findings, treatment/prevention, and future research pertaining to CTE.

## 2. Epidemiology

Concussion or mild traumatic brain injury (mTBI) is one of the most common neurologic disorders accounting for approximately 90% of all brain injuries sustained [4]. Such injuries are a common occurrence in athletes with an estimated 1.6–3.8 million sport-related concussion annually in the USA [5]. This can be seen as a gross underrepresentation of the true number as many athletes do not seek medical attention or vocalize their symptoms. This may be due to head trauma being regarded as benign, or in some the injury is not recognized at all. These behaviors are driven by the athletes' desire to return to play and the pressure to perform [6]. DeKosky et al. reported that each year more than 1.5 million Americans have mTBI with no loss of consciousness and no need for hospitalization as well as an equal number with conscious impairing trauma but insufficiently severe to require long-term hospitalization [7].

In a 2009 review of CTE, McKee et al. found that of 51 neuropathologically diagnosed cases of CTE 46 (90%) occurred in athletes. Specifically, athletes participating in American football, boxing, soccer, and hockey comprise the majority of cases. Many of these athletes began their respective sports at a young age between 11 and 19 years [5]. While it is not clear at what age CTE can begin, McKee has neuropathologically diagnosed CTE changes in asymptomatic 18-year-old high school football player with a history of concussion. While the exact incidence of CTE is unknown, it is thought to vary widely based on sport, position, length of career, number of head injuries, age of first head injury, and genetics [8].

## 3. Risk Factors

It has been well established that repetitive concussive or subconcussive blows to the head place individuals at risk for CTE [5, 6, 8]. CTE has been associated with athletes who participate in contact sports like American football, boxing, hockey, soccer, and professional wrestling. Other sports that are not directly associated with CTE, but have well-documented cases of concussion, include mixed martial arts, rugby, and horseback riding. Other groups at risk for repetitive head trauma and CTE are military veterans, epileptics, and victims of domestic abuse [5]. It has been reported that approximately 17% of professional retired boxers will exhibit CTE [3]. Although each group listed has a unifying factor of head trauma, they differ in particular aspects that may influence the severity or chronicity of their injury (see Table 1 for a summary of risk factors).

A recent study by Crisco et al. examined head impact exposure in collegiate football players and found that the average number of impacts received by an individual player during a single season was 420 with a maximum of  2,492 [9]. These impacts vary in severity based on their position. Offensive linemen, defensive linemen, and linebackers had the most frequent impacts while quarterbacks and running backs received the greatest magnitude of head impacts [9]. In the literature McKee and colleagues reported that in five football players with diagnosed CTE all played similar positions: 3 were offensive linemen, 1 defensive linemen, and one linebacker [5]. However, according to Boston University's Center for the Study of Chronic Traumatic Encephalopathy, CTE has also been found in other position players like safety and wide receiver dismissing the idea that only certain positions are at risk for developing CTE.

While it is clear that anyone who suffers repeated head trauma, regardless of the mechanism, may carry the risk of developing CTE, there is no clear consensus on how much or how little trauma is needed to cause CTE. While most feel CTE is a manifestation of repetitive trauma, the question still stands if it can be caused by a single TBI [10]. In a study by Johnson et al., widespread tau and beta-amyloid deposition was found in the brains of individuals who suffered a single traumatic brain injury. The study included the examination of postmortem brains from long-term survivors (1–47 years) of a single TBI (*N* = 39) versus uninjured age-matched controls (*N* = 47). Results showed NFTs to be exceptionally rare in young uninjured controls, yet were abundant and diffuse in one-third of TBI cases. This was also true of beta-amyloid deposition, which was found in greater density following TBI than controls [11]. While these brains showed classic changes associated with CTE, they did not have the symptomatic profile to accompany their neuropathologic findings [11]. If these subjects went on to have repeated brain injury, it would be reasonable to expect more extensive damage with more pronounced clinical symptoms.

In a study of repeated head trauma in mice, Kane et al. created an animal model where mice did not suffer severe TBI but rather mTBI to look for CTE-like changes. They reported that exposure to head trauma for 5 consecutive days showed increased expression of glial fibrillary acidic protein and phospho-tau 30 days (~160% increase) after the last injury when compared to controls. They also reported that with their mTBI model they did not find edema, cortical contusions, obvious loss of neuronal matter beneath the skull, disruption of the blood brain barrier, or microglial activation. However, they compared this to mice that were subject to a single traumatic injury and found substantial microglial activation in the hippocampus and overlying cortex 30 days after the initial impact [12].

Another high-risk group that has recently been studied are individuals in the military [12]. Operations in Iraq and Afghanistan are reporting that TBI accounts for roughly 28% of all combat casualties and approximately 88% of these are closed-head injuries [12]. While these numbers are significant, the US Defense and Veterans Brain Injury Center has estimated that approximately 180,000 soldiers have been diagnosed with mTBI between 2001 and 2010 while others estimate the number to be more than 300,000. Additionally, soldiers may also be exposed to toxins like organophosphates, chemical nerve agents, and heavy metals like uranium increasing their risk for brain injury [13].

Age is another possible risk factor for the development of CTE. At younger ages, while the brain is developing traumatic injury may begin the cascade of destructive events and compounded through the years of continued play. Conversely, at younger ages the brain has more plasticity allowing greater ability to manage injury than that in the mature brain [8, 10]. Length of play is another risk factor where longer careers with prolonged exposures to injury may cause more severe CTE. Of the 51 cases reviewed by Dr. McKee, 39 boxers had an average career of 14.4 years (range 4–25) while the 5 football players averaged careers of 18.4 years (range 14–23 years). These athletes began their respective sports between 11 and 19 years of age [5].

Genetic factors have also been thought to play a role in the development of CTE specifically the apolipoprotein E gene (ApoE). The ApoE4 allele has been well described in its association with Alzheimer's disease (AD) where individuals with homozygous ApoE4/E4 genotype have a 19-fold increased risk of developing AD [14]. This same gene is now thought to possibly have a role in the development of CTE [5]. Studies have shown that ApoE4-positive individuals had poorer outcomes with head trauma. Teasdale et al. reported that that patients with ApoE4 allele are more than twice as likely than those without ApoE4 to have unfavorable outcomes 6 months after head injury [15]. Kutner et al. examined 53 professional American football players to see if their cognitive functioning differed based on age and ApoE4 genotype. They reported that older age and presence of ApoE4 scored lower on cognitive tests than did those without the allele or with less playing experience [16]. Jordan et al. looked at ApoE4 genotype in boxers in relation to chronic TBI. They found that all boxers with severe impairment, based on the chronic brain injury scale, had at least 1 ApoE4 allele. Therefore, they reported that ApoE4 may be associated with increased severity of chronic neurologic deficits in high-exposure boxers [17].

In McKee and colleagues' review of the 51 CTE cases, ApoE genotyping was reported in 10 cases where 50% carried at least one ApoE4 allele and one was homozygous for E4. While they did not report what the other 4 genotypes were, it raised their suspicions to believe that ApoE4 was the gene of interest. In animal studies ApoE4 transgenic mice had greater mortality from TBI than those with ApoE3 allele. Another study showed that transgenic mice that overexpress ApoE4 allele showed increased deposition of beta-amyloid after experimental TBI [5].

However, a study by Omalu et al. reported that 70% of their CTE cohort had the ApoE3 genotype. Of the 17 athletes they used in their study they were able to determine the ApoE genotype in 10 of 14 professional athletes and in 2 of 3 high school athletes. Of the 10 professional athletes 90% had at least one ApoE3 allele, and 7 of the 10 with confirmed ApoE genotype also had CTE. Of these 7 athletes with CTE 100% had at least one ApoE3 allele (5 E3/E3, 2 E3/E4). It should also be noted that the only professional athlete in their study that did not have the E3 allele (E2/E4) was negative for CTE. Additionally, the two other professional athletes that had the ApoE allele but did not have CTE were E2/E3 (24 years old) and E3/E3. The authors note that the one case of E3/E3 that did not have tauopathy was assessed from only select sections of the brain as they did not have access to the full brain. Of the two high school athletes both were E3/E3 genotypes and did not show any histological signs of CTE [18].

## 4. Clinical Presentation

It is important to define the timeline of CTE symptom development to distinguish it from concussive or postconcussive syndrome (PCS). The symptoms associated with acute concussion are headache, blurred vision, amnesia, tinnitus, fatigue, and slurred speech with resolution within days to weeks if managed properly. Although there is no strict timeline to the acute injury phase, it has typically been reported as three months, but 80–90% of patients show full recovery within the first 10 days [19]. When symptoms extend beyond three months, the individual is said to have PCS. These individuals will have additional symptoms of physical, emotional, cognitive, and behavioral problems [6]. PCS also has a variable timeline where symptoms typically improve within one year but may in some cases require several years for resolution. For those with persisting or permanent PCS symptoms, they would logically be considered to have CTE.

However, the typical clinical course of an individual who develops CTE is not as linear as direct progression from concussion to PCS to CTE. The onset of CTE symptoms typically starts later in the lives of certain athletes after the individual has removed themselves from competition. As reported by McKee, the first symptoms of CTE were noted at ages ranging from 25 to 76 years. McKee also reported that at the time of retirement one-third of these athletes were symptomatic with another half becoming symptomatic within 4 years of leaving sports [5]. In 14 cases (30%), mood disturbances were reported while movement abnormalities like Parkinsonism, ataxia, antalgic gait, and dysarthric speech were reported in 41% of  subjects [5]. The course of symptom progression seems to follow a somewhat continuous path beginning with cognitive and emotional decline leading to eventual motor deterioration [5].

Initially, patients begin to have poor concentration, attention, and memory along with disorientation, dizziness, and headaches. They typically progress to experience irritability, outbursts of violent or aggressive behavior, confusion, and speech abnormalities. During this stage of the disease, there is a high frequency of suicide, drug overdose, and mood disorders, mainly major depressive disorder [5]. A study by Omalu and colleagues describes a similar clinical profile with a latent asymptomatic period between play and symptom onset. He reports worsening of cognitive and social functioning leading to poor money management, bankruptcy, social phobias, paranoid ideation, insomnia, poor relationships, divorce, emotional/physical abuse, and substance abuse [18]. Family and friends of the affected individuals reported many of these symptoms to researchers through standard forensic interviews [18].

As the disease progresses in severity, there is a greater loss of motor functioning. Some patients may develop Parkinsonian symptoms of tremors, masked facieses, wide propulsive gait, poor speech, ocular abnormalities, vertigo, bradykinesia, deafness, and a small group developing dementia. Currently, the number of cases with confirmed dementia remains small. As more postmortem exams are done in the at risk group, it is expected more cases will be diagnosed. Some individuals with CTE have committed suicide, overdosed on drugs, or died from accidents preventing progression of the disease [5, 20].

## 5. Pathophysiology

The development of CTE is due to repetitive traumatic brain injury from the acceleration/deceleration forces of closed head impacts [5]. Damage to the brain generally occurs when the brain collides against the skull causing damage to the same side as the collision, coup, or to the opposite side of the impact, countercoup [6]. High-speed decelerations may also cause mechanical and chemical injury to the long axons resulting in traumatic/diffuse axonal injury. Crisco et al. report that impacts to the top of the head had the lowest rotational force but highest linear force leading more to cervical spine injuries. However, lateral blows cause rotational forces, which are the typical cause of mild traumatic brain injury [6]. While there is a firm understanding of head trauma being the general cause of the brain damage in CTE, researchers have not agreed upon a unifying mechanism of injury. Originally the associated axonal damage was thought to be due to shearing or mechanical forces at the time of injury. However, it is now reported that axonal shearing or tearing is a secondary event to the acute inflammation and neurodegeneration of axons [5]. In the acute setting there is rapid axonal swelling, perisomatic axotomy, and Wallerian degeneration [5].

Gavett et al. offered a general description of how the damage may occur through the repeated traumatic injury of axons. Damage to axons would cause changes in membrane permeability and ionic shifts causing a large influx of calcium. Subsequent release of caspases and calpains would trigger tau phosphorylation, misfolding, shortening, and aggregation as well as cytoskeleton failure with dissolution of neurofilaments and microtubules [8].

This idea was elaborated on in a recent paper by Blaylock and Maroon who describes the concept of immunoexcitotoxicity as a possible central mechanism for CTE. He describes a cascade of events that begin with an initial head trauma, which “primes” the microglia for subsequent injuries. When the homeostasis of the brain is disturbed, some of the microglia undergo changes to set them in a partially activated state. When these microglia become fully activated by continued head trauma, they release toxic levels of cytokines, chemokines, immune mediators, and excitotoxins like glutamate, aspartate, and quinolinic acid. These excitotoxins inhibit phosphatases, which results in hyperphosphorylated tau and eventually neurotubule dysfunction and neurofibrillary tangle deposition in particular areas of the brain [10]. There is also an apparent synergy between the proinflammatory cytokines and glutamate receptors that worsen neurodegeneration in injured brain tissue. This combination also increases the reactive oxygen and nitrogen intermediates that interfere with glutamate clearance keeping the injury response high. Priming can also occur from insults to the brain like systemic infections, environmental toxins, and latent viral infections in the brain (cytomegalovirus and herpes simplex virus) [10].

The microglia, however, have a dual function allowing them to switch between being neurodestructive and neuroreparative. During acute injury the microglia are responsible for containing the damage with inflammation, cleaning up debris, and repairing the surrounding damaged tissue [10]. However, if the individual experiences a second brain trauma or multiple continuous traumas, the microglia may never have the chance to switch from proinflammatory to reparative mode [10]. Such repetitive trauma may place the brain in a state of continuous hyperreactivity leading to progressive and prolonged neuronal injury. This would support the evidence that repeated mTBI results in a higher incidence of prolonged neurological damage than single-event injury [10].

The eventual neurodegeneration is also dependent on other factors like the age of the brain at the time of injury. Several studies have shown that older individuals have poorer outcomes when compared to younger subjects experiencing TBI. Streit et al. showed that as the microglia age, they become more dysfunctional, which may impair their ability to terminate immune activation. Therefore, as the brain grows older, it has more activation of microglia with weaker mitochondrial functioning, neuronal and glial dystrophy, higher levels of inflammation, and lifetime exposures to environmental toxins [10].

## 6. Gross Pathology Findings

As described by Corsellis et al., the most common gross pathological findings in CTE included reduced brain weight, enlargement of the lateral and third ventricles, thinning of the corpus callosum, cavum septum peallucidum with fenestrations, scarring, and neuronal loss of the cerebellar tonsils. Brain atrophy was most severe in the frontal lobes (36%), temporal lobes (31%), and parietal lobes (22%) with the occipital lobe rarely being affected [5]. McKee and colleagues reported that with increasing severity of disease marked atrophy is noted in the hippocampus, entorhinal cortex, and the amygdala [5]. Blaylock and Maroon reported that these areas showed the most severe atrophy and were noted to have the highest concentration of glutamate receptors and cytokine receptors [10].

## 7. Microscopic Pathology

According to Dr. Bennet Omalu, a forensic neuropathologist, the basic feature of CTE is the presence of sparse, moderate, or frequent band-shaped, flame-shaped small globose and large globose neurofibrillary tangles (NFTs) in the brain accompanied by sparse, moderate, or frequent neuropil threads (NTs) [18]. Similarly, McKee and colleagues described the core pathology of CTE to include tau-reactive NFTs, astrocytic tangles, and dot-like spindle-shaped NTs [10]. These changes are commonly noted in the dorsolateral frontal, subcallosal, insular, temporal, dorsolateral parietal, and inferior occipital cortices. Additionally, Dr. McKee also reported occasional tau immunoreactive neuritis and NFTs in the posterior, lateral, and/or anterior horns of the spinal cord (see Table 2) [10].

Beta-amyloid (A*β*) deposition is an inconsistent finding in CTE as Dr. McKee noted that of the 51 cases of confirmed CTE she reviewed that only 3 (6%) had amyloid angiopathy [5]. Animal studies by Iwata et al. used swine TBI models to show minimal A*β* accumulation in axons acutely after injury but saw greater accumulation one month following injury [20]. Similarly Chen et al., also using a swine TBI model, found evidence of axonal pathology 6 months following rotational brain injuries [21].

Although CTE and Alzheimer's disease (AD) both have NFTs and possibly beta-amyloid plaques, there are several unique features that distinguish the two [8]. First, beta-amyloid deposits are only found in 40% to 45% of patients with CTE while they are present in nearly all cases of AD. Secondly, the tau distribution in CTE is located more in the superficial cortical laminae whereas in AD they are found in large projection neurons in deeper layers. It is also important to note is the distribution of the NFTs in CTE that extremely irregular with uneven foci in the frontal temporal and insular cortices, while AD has a more uniform cortical NFT distribution. DeKosky et al. noted that the hippocampus is frequently spared by tauopathy in CTE, whereas it is the first location affected by tauopathy in AD [7]. Lastly, NFTs in CTE are most concentrated at the depths of the cortical sulci and are typically perivascular, which might indicate that there are disruptions of cerebral microvasculature and the blood-brain barrier at the time of injury leading to NFT formation [8].

A recent study by Omalu et al. described four histomorphologic phenotypes of CTE in American athletes (see Table 3). They examined specimens from 17 deceased athletes, 10 of which had histopathologically confirmed CTE. All were male, age range of 17–52, (8 were American football players, 4 professional wrestlers, 1 mixed martial arts fighter, 1 professional boxer, and 3 high school American football players). Omalu and colleagues created these histologic phenotypes based on the presences or absence of NFTs, NTs, and diffuse amyloid plaques as well as their quantitative distribution in the cerebral cortex, subcortical nuclei/basal ganglia, hippocampus, and cerebellum. These results are summarized in Tables 1 and 2. Phenotype one shows sparse to frequent NFT and NTs in the cerebral cortex and brainstem without involvement of the subcortical nuclei, basal ganglia, or cerebellum without any beta-amyloid. Phenotype two is the same as the first except they show diffuse beta-amyloid plaques. The third group had higher concentrations of NFTs and NTs only in the brainstem without involvement elsewhere with no beta-amyloid. The fourth group had sparse NFTs and NTs in the cerebral cortex, brainstem, subcortical nuclei, and basal ganglia with an unaffected cerebellum and no beta-amyloid [18].

Trans-activator regulatory DNA-binding protein 43 or TDP-43 has been a recent addition to the growing neuropathologic findings associated with CTE. TDP-43 is a highly conserved protein that is found in many tissues including the CNS [22]. It plays a significant role in mediating the response of the neuronal cytoskeleton to axonal injury [8]. In a study by McKee et al., they reported widespread TDP-43 proteinopathy in 80% of their CTE cases. Until this study, TDP-43 was thought to be a unique finding to amyotrophic lateral sclerosis (ALS) and frontotemporal lobar degeneration (FTLD-TDP) but has now been found in other neurodegenerative diseases as a secondary pathology [22].

## 8. Clinicopathologic Correlations

The typical symptoms of CTE can be directly connected to the specific areas of the brain that are injured during the progression of disease. Based on these symptoms it is clear that there is damage to the hippocampal-septo-hypothalamic-mesencephalic circuitry (Papez circuit) also known as the emotional or visceral brain [5]. Damage to these areas correlate to the behavioral symptoms of emotional liability, aggression, and violence. Damage to the hippocampus, enteorhinal cortex, and medial thalamus conceivably causes the commonly reported complaint of memory disturbance. Destruction to the frontal cortex and white matter may result in the dysexecutive symptoms found throughout the many cases of CTE. Motor abnormalities may be due to degeneration of the substantia nigra and pars compacta along with symptoms of dysarthria, dysphagia, and ocular malfunction due to brainstem nuclei injury like the hypoglossal and oculomotor nuclei (see Table 4) [5].

## 9. Neurological Sequelae

Historically amyotrophic lateral sclerosis (ALS) has been thought to be a sporadic disease with no single causative factor. Literature has reported risk factors to include trauma to the brain or spinal cord, strenuous physical activity, exposure to heavy metals, cigarette smoking, radiation, electrical shocks, and pesticides [22]. Given these risk factors, the literature strongly correlates a history of head trauma with increased incidence of ALS. In a case control study, Chen et al. reported that having repeated head trauma within the 10 years prior to diagnosis had a 3-fold higher risk of ALS [23]. The same group also did a meta-analysis of 8 ALS studies and estimated a pooled odds ratio of 1.7 (95% CI: 1.3, 2.2) for at least one previous head injury. Another study reported increased ALS incidence and mortality in professional Italian soccer players when compared to the general population [24]. Additionally, an incidence study of 7,325 Italian professional soccer players showed an ALS incidence 6.5 times higher than expected [25]. ALS has also been seen in higher numbers among American and Canadian football players when compared to the general population [22]. The risk of ALS has also been reportedly high in war veterans. A study of Gulf War veterans reported that the risk of ALS was increased 2-fold during the 10 years following service [26]. A study by Schmidt et al. reported that veterans who received head trauma during war had an adjusted odds ratio for the development of ALS of 2.33 (95% CI: 1.18–4.61) [27].

 A recent study by McKee and colleagues examined and compared the brains and spinal cords of 12 athletes with confirmed CTE to 12 cases of sporadic ALS to12-age matched controls. Of the 12 CTE cases, 3 also had a diagnosed motor neuron disease (MND) resembling ALS. The study found that those with CTE and the motor neuron disease not only had the typical neuropathologic presentation of CTE with tau-NFT, NT, and TDP-43 throughout the brain and brain stem, but they also had these changes in the anterior horns of the spinal cord in high concentrations. Of the 9 CTE patients that did not have the MND, they had similar CTE neuropathology, but it did not affect the spinal cord as significantly. When compared to the samples of sporadic ALS, they found TDP-43 immunoreactivity in all 12 cases with no tau immunoreactive NFTs. The age-matched controls showed no TDP-43 or tau reactivity. These results indicate that the widespread tauopathy and TDP-43 proteinopathy of CTE can in some cases extend beyond the brain and the brain stem to severely affect the spinal cord. The authors have labeled these cases as having chronic traumatic encephalomyelopathy (CTEM). While this has only been identified in three cases, it opens the floor to further discussion and research to see if CTEM is in fact a unique disease or just the coincidental occurrence of ALS and CTE. McKee has stated that the tau pathology in the three cases of CTEM is not only distinct from that of sporadic ALS, but the nature and distribution of the TDP-43 proteinopathy are also unique [22].

## 10. Diagnosis

Currently the only way to definitively diagnose CTE is through postmortem neuropathological autopsy [8]. Clinical diagnosis is difficult due to a lack of consensus on diagnostic criteria or large-scale longitudinal clinicopathologic correlation studies [8]. The differential diagnosis for CTE usually includes diseases like AD and frontotemporal dementia (FTD), which all share similar clinical symptoms, and all may have a history of head trauma making a clinical diagnosis difficult. Although age can help in distinguishing between AD and CTE, it does not help when deciding between FTD and CTE.

It is the hope of many that the advances in neuroimaging will aid in detecting chronic and acute changes associated with CTE. Diffusion tensor imaging (DTI) has been reported to be sensitive enough to assess axonal integrity in the setting of mild, moderate, and severe TBI. DTI studies have shown their ability to show occult white matter damage after mTBI that was not visible on typical MRI scans. A study by Kumar et al. tracked serial changes in white matter using DTI techniques in mTBI and found that fractional anisotropy (FA) and mean diffusivity (MD) in the genu of the corpus callosum appear early and persisted at 6 months as a secondary injury to microgliosis [28]. Another study by Inglese et al. showed the abilities of DTI as they reported significant abnormalities in various regions of the brain after mTBI when compared to controls [29]. A third study by Henry and colleagues used DTI to detect changes in white matter by comparing a group of 10 nonconcussed athletes to 18 concussed athletes. They reported that at 1–6 days and at 6 months following concussion there was FA in dorsal regions of both cortical spinal tracts and the corpus callosum [30].

Although researchers are trying to identify biomarkers to aid in diagnosis, there currently are no makers identified in the literature that can be used to diagnosis CTE. However, there are several that are believed to help in identifying CTE like the use of magnetic resonance spectroscopy that can detect changes in glutamate/glutamine, N-acetyl aspartate, and mylo-inositol which have been shown to be abnormal in brain injury [8]. There has also been discussion of attempting to measure tau and phospho-tau in the cerebrospinal fluid of those suspected of having CTE [8].

## 11. Treatments/Prevention

Currently, the treatment methodologies for CTE are purely preventive. However, in sports like American football, prevention of head trauma is a seemingly difficult goal to attain. Hard hits and head collisions are more than simple aspects of the game; they are part of the sports identity. Therefore, prevention would require a multifaceted approach involving administrators, coaches, players, referees, team physicians, and even the fans who watch the games. The administrators create the policies that penalize athletes for reckless or dangerous hits as well as setting equipment standards for the various leagues. It is the role of the coaches to teach their players correct and safe technique for tackling, hitting, and personal protection while creating a team culture that encourages hard but controlled play. Coaches also need to be aware of the cumulative effect of repetitive mTBI and limit the amount of full contact during practice and drills. It is the role of the players to understand the potential dangers and consequences of head trauma beyond their playing years so they can protect themselves and limit the number of injuries during their career. It is also incumbent on the athlete to not downplay their injuries and to seek help or advice if they are suffering from signs or symptoms of head trauma. As for the referees, it is their role to create a safe playing environment and uphold the rules set forth to protect the players whether on the field, in the ring, or on the ice. As for the team physician, it is their task to remove players from play and appropriately manage their mTBI until they meet the return-to-play criteria. The decision to clear a player is challenging for the physician who has no baseline information of the player's cognitive function prior to the injury. Therefore, it has been suggested that players undergo neuropsychological testing prior to participation in sports as a tool to properly assess the athlete's cognitive deficits both acutely and chronically.

Another aspect of prevention is improving the protective equipment worn by the athletes. It has been shown that helmets and mouth guards function very well in protecting the player from severe head injury if the helmet fits correctly, is strapped in place, and lined with the appropriate padding [6]. A study by Viano and Halstead compared American football helmets from 1970 to 2010 and reported that the newer helmets are heavier, primarily from more padding, longer, higher, and wider then their 1970s counterparts. These larger helmets were better at absorbing forces and impacts associated with concussions in American football [31]. While helmets are important, they may also give some players a false sense of protection leading to a more reckless and violent style of play. Neck strength is another factor that can be important in minimizing head injury especially in younger populations of athletes and should be emphasized by trainers and strength coaches [6]. Some groups are looking for medical therapies to limit the damage after a head injury. Particularly, the use of beta-amyloid-lowering medications has been shown to improve the outcomes following TBI in rodent models [7].

## 12. Future Considerations for Research

Although there has been an exponential growth in research and interest in CTE over the last five years, the full understanding of it still remains in its infancy. Affected athletes have been the greatest supporters as Boston University's Center for the Study of CTE has more than 260 former athletes in their brain and spinal cord donation registry. These donations will supply researchers with a wealth of information that will improve animal models, better define the mechanism of injury, as well as advance diagnosis and treatment. As the foundation of knowledge grows, we can better identify genetic variants that put individuals at risk for CTE. The CTE community will also benefit from ongoing concussion research as groups look for acute biomarkers to be used as a diagnostic test for brain injury. There is also a need for further research regarding the role of advanced neuroimaging like DTI and its ability to possibly detect early signs of acute injury. Additionally, more work must be done to quantify the magnitude and frequency of head impact that is needed to cause the neurodegeneration associated with CTE.

## 13. Conclusions

Chronic traumatic encephalopathy is a neurodegenerative disease that is a long-term consequence of single or repetitive closed head injuries for which there is no treatment and no definitive premortem diagnosis. It has been closely tied to athletes who participate in contact sports like boxing, American football, soccer, professional wrestling, and hockey. Aside from repeated head trauma, risk factors include presence of ApoE3 or ApoE4 allele, military service, and old age. It is histologically identified by the presence of tau-immunoreactive NFTs and NTs with some cases having a TDP-43 proteinopathy or beta-amyloid plaques. It has an insidious clinical presentation that begins with cognitive and emotional disturbances and can progress to Parkinsonian symptoms. The exact mechanism for CTE has not been precisely defined; however, research suggests it is due to an ongoing metabolic and immunologic cascade called immunoexcitotoxicity. Current research is attempting to identify specific biomarkers along with more sophisticated imaging techniques for the diagnosis of CTE. Future research should also be centered around how to manage CTE as suicide is a common fate for those battling the disease. Further efforts need to be made to educate players, coaches, and administrators of all levels of athletics to make them aware of the determents of mTBI and how to best protect themselves. There must also be further investigations into the possible link between CTE and motor neuron disease. Establishing such a causal link may open new doors in ALS research and hopefully lead to better treatments. Through the continued efforts of athletes, scientists, and physicians, our knowledge of CTE will advance and allow for the evolution of better diagnosis, treatment, and prevention.

## Figures and Tables

**Table 1:** Risk factors associated with chronic traumatic encephalopathy.

(i) Head trauma: single or repetitive	
(ii) History of head concussion	
(iii) Participation in the following	
Boxing	
American football: offensive/defensive linemen/linebackers/running backs	
Soccer	
Professional wrestling	
Hockey	
Military service: blast injuries	
(iv) Length of sport participation	
(v) Epileptics	
(vi) Victims of domestic abuse	
(vii) Age of injuries: younger ages and older ages	
(viii) Genetic variation: ApoE3 or ApoE4	

**Table 2:** Areas of damage in the brain.

*Gross areas of damage *	
(i) Reduced brain weight with atrophy of	
Frontal lobe	
Temporal lobe	
Parietal lobe	
Occipital lobe	
(ii) Enlargement of lateral and third ventricles	
(iii) Thinning of the corpus callosum	
(iv) Cavum septum pellucidum with fenestrations	
(v) Scarring and neuronal loss of cerebellar tonsils	
(vi) Pallor of substantia nigra	

* Areas of tau NFTs and NT *	
(i) Superficial cortical layers	
(ii) Dorsolateral frontal	
(iii) Subcallosal	
(iv) Insular	
(v) Temporal	
(vi) Dorsolateral parietal	
(vii) Inferior occipital cortices	
(viii) Thalamus	
(ix) Hypothalamus	
(x) Substantia nigra	
(xi) Olfactory bulbs	
(xii) Hippocampus	
(xiii) Entorhinal cortex	
(xiv) Amygdala	
(xv) Brainstem	

**Table 3:** Emerging histomorphologic phenotypes in American athletes.

Phenotype	Histological findings
1	Sparse to frequent NFT and NTs in the cerebral cortex and brainstem without involvement of the subcortical nuclei, basal ganglia, or cerebellum without any beta-amyloid
2	Sparse to frequent NFT and NTs in the cerebral cortex and brainstem with diffuse beta-amyloid deposition. No involvement of the subcortical nuclei, basal ganglia, or cerebellum
3	Higher concentrations of NFTs and NTs only in the brainstem. No involvement elsewhere or any beta-amyloid
4	Sparse NFTs and NTs in the cerebral cortex, brainstem, subcortical nuclei, and basal ganglia with an unaffected cerebellum and no beta-amyloid [18]

**Table 4:** Clinicopathological correlations [5].

Damage area	Clinical presentation
Hippocampus Entorhinal cortex Medial thalamus	Early deficits in memory

Frontal cortex and underlying white matter	Dysexecutive symptoms

Dorsolateral parietal Posterior temporal Occipital cortices	Visuospatial difficulties

Substantia nigra Pars compacta	Parkinsonian motor features

Cortical and subcortical frontal damage Cerebellar tract injury in brainstem	Gait disorder: staggered, slowed, ataxic

Brainstem nuclei (hypoglossal/oculomotor)	Dysarthria, dysphagia, ocular abnormalities

Amygdala	Aggression and violent outbursts
